# IL-17A and TNF-α Increase the Expression of the Antiapoptotic Adhesion Molecule Amigo-2 in Arthritis Synoviocytes

**DOI:** 10.3389/fimmu.2016.00254

**Published:** 2016-06-27

**Authors:** Giulia Benedetti, Paola Bonaventura, Fabien Lavocat, Pierre Miossec

**Affiliations:** ^1^Immunogenomics and Inflammation Research Unit EA 4130, Department of Clinical Immunology and Rheumatology, Edouard Herriot Hospital, University of Lyon 1, Lyon, France

**Keywords:** rheumatoid arthritis, amigo-2, IL-17A, TNF-α, HMGB1, ERK, synoviocyte survival and proliferation

## Abstract

Rheumatoid arthritis (RA) is a chronic inflammatory disorder, characterized by a persistent immune cell infiltrate in the synovium accompanied by high levels of inflammatory mediators and synovial hyperplasia. Despite significant therapeutic advances, RA remains an important unmet medical need. To discover potential new genes controlling inflammation and apoptosis in synoviocytes, genes induced by the two pro-inflammatory cytokines, tumor necrosis factor α (TNF-α) and interleukin 17A (IL-17A), were systematically searched. We identified Amphoterin-induced gene and ORF 2 (Amigo-2), a novel antiapoptotic adhesion molecule, as synergistically upregulated by the IL-17A/TNF combination specifically in RA synoviocytes. In addition, when RA synoviocytes were cocultured with immune cells, Amigo2 expression was significantly increased in both fibroblasts and immune cells. This induction persisted in RA synoviocytes even after the removal of the immune cells. Amigo2 induction was ERK-dependent and on the contrary, inhibited by JNK. Furthermore, Amigo2 expression levels correlated with apoptosis of the cells when exposed to the proapoptotic agent cadmium (Cd). Interestingly, exposure of the cells to HMGB1 in inflammatory conditions increased synergistically Amigo2 expression and significantly reduced Cd-mediated cellular toxicity. Our findings support a model whereby cell–cell contact with immune cells and exposure to the combination of both inflammatory cytokines and HMGB1 in the joints of RA patients increases Amigo2 expression in synoviocytes in an ERK-dependent manner which, in turn, enhances cellular adhesion and promotes cell survival and cellular proliferation.

## Introduction

Rheumatoid arthritis (RA) is a chronic inflammatory disease characterized by synovium hyperplasia leading to progressive joint destruction and bone resorption (1, 2). The synoviocytes present in the synovial intimal lining are key contributors to RA pathogenesis. They produce cytokines that perpetuate inflammation and secrete proteases contributing to cartilage destruction. Their excessive proliferation and apoptosis resistance are the cause of the synovial hypertrophy and their migratory and invasive properties exacerbate joint damage (3). So far, treatment of RA is based on targeting the immune system with no direct effect on synoviocytes. Removing the hypertrophic pathologic synovial tissue by surgical, chemical, or radiation relieves arthritis for a more prolonged time but is complex to use in a polyarticular situation (4, 5). Therefore, new molecules controlling synovial hyperplasia need to be discovered for the development of more synoviocyte-targeted therapeutic solutions.

The two pro-inflammatory cytokines tumor necrosis factor α (TNF-α) and interleukin 17A (IL-17A) are important contributors to RA chronicity. They both induce the production of several inflammatory mediators in the diseased synovium. Furthermore, these two cytokines synergize to induce several antiapoptotic molecules in RA synoviocytes (6–10) and a large amount of neutrophilic mediators, perpetuating the primary inflammatory response (7, 10–13). To discover new apoptosis and inflammatory regulators in RA synoviocytes, we searched for genes induced by the pro-inflammatory cytokines TNF-α and IL-17A in a previously performed 12-h transcriptomics analysis. We identified Amphoterin-induced gene and ORF 2 (Amigo-2), which was synergistically up-regulated by the IL-17A/TNF combination.

Amigo2, also known as Alivin-1, is part of a novel family of genes encoding for type I transmembrane proteins with two other members, namely AMIGO and AMIGO-3. All AMIGOs share a similar protein structure composed of an extracellular domain containing six leucine-rich repeats (LRRs) mediating cell–cell interaction followed by an immunoglobulin domain, a transmembrane domain and an intracellular domain with several possible phosphorylation sites. They form homo- and heterodimers and potentially lead to signal transduction in the cells (14, 15). Interestingly, AMIGO-2 was shown to inhibit apoptosis and to promote the survival of electrical active neuronal cells (16). AMIGO-2 also increased the migration and invasion capacities of gastric cancer cells. Stable AMIGO-2 knockdown in gastric adenocarcinoma cells affected the morphology, the ploidy, the chromosomal stability and the cellular adhesion and migration of the cells and almost completely abrogated tumorigenicity in nude mice (17). In addition, the closely related family member AMIGO was found to be induced by the heparin-binding protein amphoterin, also known as HMGB1 (14). Excessive extracellular HMGB1 levels have been detected in joints and serum of RA patients with higher levels found in the regions where proliferating synovial tissue invaded cartilage and bone. Furthermore, direct injection of HMGB1 into murine knee joints initiated persistent inflammatory responses and synovitis and antagonistic HMGB1 therapies ameliorated arthritis (18).

In this study, we showed that the IL-17A/TNF combination synergistically increased Amigo2 expression specifically in RA synoviocytes. Amigo2 was also upregulated when RA synoviocytes were cocultured with peripheral blood mononuclear cells (PBMC) in both cell types and this induction persisted in RA synoviocytes even after the removal of the immune cells. Furthermore, we demonstrated that the IL-17A/TNF-mediated Amigo2 induction was promoted by ERK and by HMGB1 but was inhibited by JNK. In addition, Amigo2 expression levels correlated with apoptosis of the cells when exposed to the proapoptotic agent Cd- and HMGB1-mediated Amigo2 induction protected the cells against Cd toxicity in inflammatory conditions. In conclusion, our study showed, for the first time, that cell–cell contact with immune cells and exposure to the combination of both inflammatory cytokines and HMGB1 increases the expression of the gene Amigo2 in RA synoviocytes, which promotes cell survival.

## Materials and Methods

### Isolation and Culture of Synoviocytes

Synoviocytes were grown from synovial tissue samples obtained from healthy donors or patients suffering from osteoarthritis or RA undergoing joint surgery. Each individual signed an informed consent and the protocol was approved by the committee for protection of persons participating in biomedical research under the number AC-2010-11-64. The RA patients fulfilled the American College of Rheumatology criteria for RA (19). Briefly, synovial tissue was minced in small pieces which were allowed to adhere on plastic plates. Those samples were maintained in DMEM medium (Eurobio, Courtaboeuf, France) supplemented with 10% FBS (Life Technologies), 2% Penicillin–Streptomycin (Eurobio), 1% l-glutamine (Eurobio), and 1% Amphotericin B (Eurobio) until cells grew out of the tissue and colonized the plastic dishes. After cells reached confluence, tissue pieces were removed, and cells were trypsinized. Synoviocytes were used between passages 4 and 9.

### Culture Conditions

Cells were exposed to IL-17A 50 ng/mL (R&D systems, Minneapolis, MN, USA), TNF-α 0.5 ng/mL (R&D systems), or a combination of both cytokines. Exposure to the apoptotic agent, cadmium 0.1 ppm (kindly provided by Pr. Albarède, Geology laboratory, ENS, Lyon, France), was performed after treating the cells overnight with a combination of IL-17A and TNF-α or with the vehicle. HMGB1 exposure at 50 ng/mL (R&D systems) was done in the presence or not of the combined IL-17A/TNF-α treatment. Exposures to the mitogen-activated protein kinases (MAPKs) inhibitors SP6000125 30 μM (Calbiochem), SB203580 5 μM (Calbiochem), and U0126 50 μM (Calbiochem) were done 1 h prior cytokine addition. Cells were treated for 12 or 6 h for mRNA extraction or several days for the functional assays.

### Cocultures of Synoviocytes and Peripheral Blood Mononuclear Cells

Peripheral blood mononuclear cells from healthy donors were separated by Ficoll-Hypaque density-gradient centrifugation. PBMC were cultured in complete RPMI medium (Eurobio) supplemented with 10% AB-human serum (Invitrogen, Saint Aubin, France), 2% Penicillin–Streptomycin (Eurobio), and 1% l-glutamine (Eurobio). Cocultures were performed in flat-bottomed 96-well culture plates in which 10^4^ synoviocytes/well were seeded and allowed to adhere to the plate for several hours. PBMC (5 × 10^4^) were then added to the synoviocytes in the presence or not of 5 ug/mL phytohemagglutinin (PHA, Sigma Aldrich, Saint-Quentin Fallavier, France). After 24 h, supernatants were collected and cells were washed with PBS, followed by partial separation of PBMC from synoviocytes by incubating the cocultures with a 1 mM EDTA–PBS solution at 37°C for a brief time. PBMC present in the EDTA wash and synoviocytes still attached on the plate layer were then lysed for the 24 h timepoint. Complete RPMI medium in the presence or not of PHA was then added to the cells for 24 or 48 h more before collection of the supernatants and lysis of the cells. For each condition, twelve wells were combined in order to obtain sufficient material to perform gene expression quantifications.

### Cell Death Assays

Cell death was determined by flow cytometry and cell cycle analysis. Cell cycle analysis was performed by fixating the cells in ethanol during at least 24 h and by subsequent staining of the cells with 3.3 μM of 4,6-diamidino-2-phenylindole (DAPI). Fluorescence was analyzed on a Navios flow cytometer (Beckman Coulter, Brea, CA, USA), and the amount of dead cells in sub-G0/G1 was determined using the Kaluza® flow analysis software (Beckman Coulter, Brea, CA, USA).

### Quantitative Real-time PCR

Total RNA was isolated from cells using an RNeasy® Mini kit (Qiagen, Hilden, GE) according to the manufacturer’s protocol. cDNA was synthesized using the QuantiTect reverse transcription kit (Qiagen) following manufacturer’s instructions. Reactions were performed on the CFX96 Real-Time PCR Detection System (Biorad, Marnes-la-Coquette, France) using the QuantiFast SYBR green kit and the Qiagen QuantiTect primers (QT01034817 for Amigo2, QT00025011 for Bcl2, QT00001792 for CD3 and QT01192646 for GAPDH). Cycle threshold (Ct) values were normalized to the expression levels of the housekeeping gene GAPDH. The relative expression of the genes in treated cells versus control cells was determined using the ΔCt method.

### ELISA

Tumor necrosis factor α and IL-17A production (R&D system) were quantified in the supernatants of exposed synoviocytes or of cocultures after the indicated culture duration and according to the manufacturer’s instructions. Absorbance at 450 nm was measured using the multilabel plate reader VICTOR™ X4 (by Perkin Elmer, Waltham, MA, USA), and results were obtained by subtracting the background read at 540 nm.

### Statistical Analyses

All data are expressed as mean ± SEM. RT-PCR data were log2 transformed before statistical analyses. Statistical significance was determined by GraphPad Prism using a two-way Annova with Bonferroni posttests. The level of confidence is represented by *P*-values indicated in the figures.

## Results

### Amigo2 Is Upregulated More Specifically in RA Synoviocytes in Inflammatory Conditions

**Figure 1:**
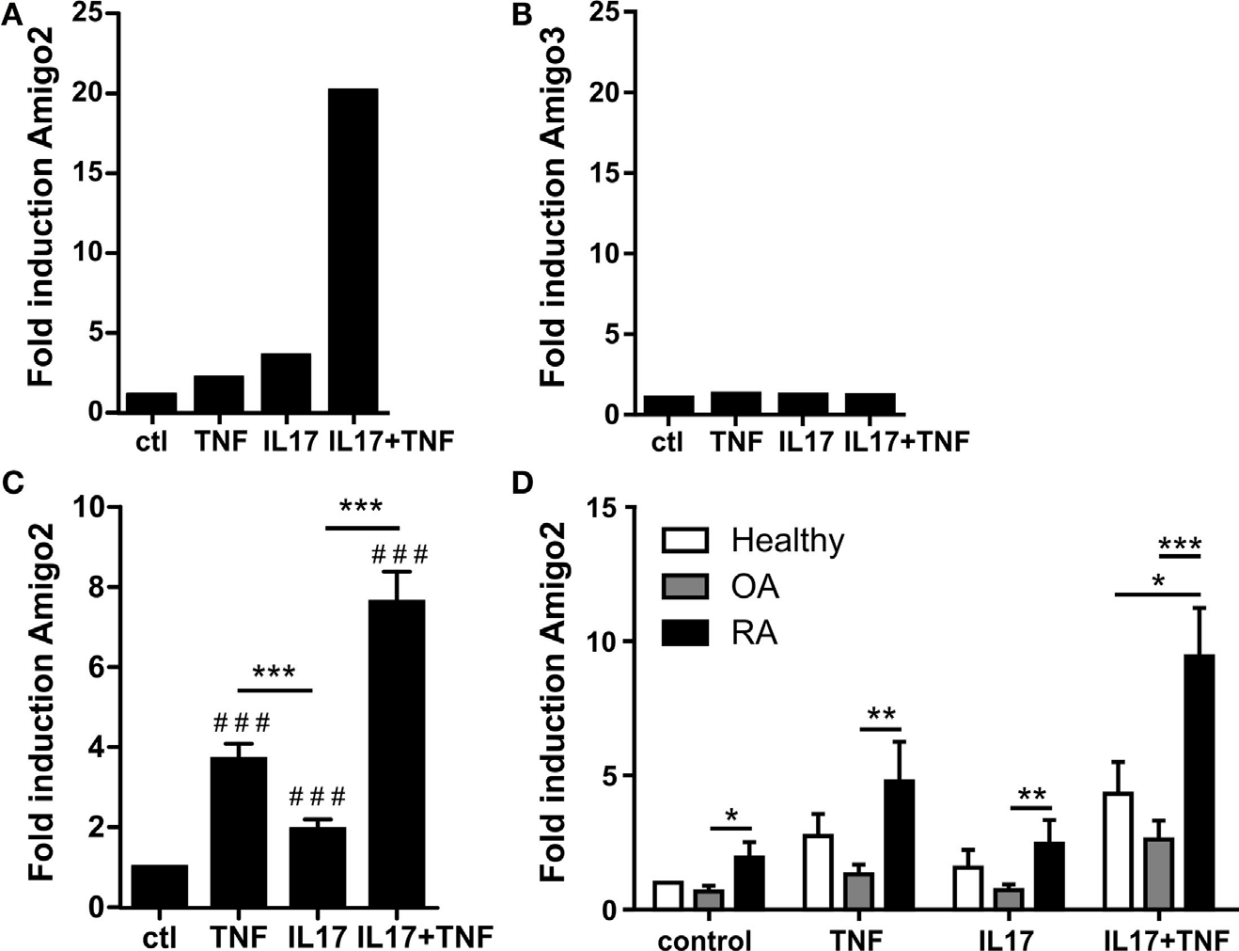
**Amigo2 is upregulated more specifically in RA synoviocytes in inflammatory conditions**. Synoviocytes were exposed to IL-17A or TNF-α alone or to a combination of both for 12 h. Amigo2 and Amigo3 expressions were assessed by transcriptomic analysis **(A,B)** and Amigo2 expression was also assessed by quantitative real-time PCR **(C,D)**. Gene expression was compared between the different treatments and is represented as fold changes compared to control in RA synoviocytes **(A–C)**. Amigo2 expression was also evaluated in synoviocytes from different clinical settings (healthy, OA and RA) and is expressed as fold changes compared to healthy synoviocytes exposed to vehicle **(D)**. Data are the mean of at least three independent experiments ± SEM. ^#^Comparison with control situation, *comparison between different cytokine combinations **(C)** or between cell types **(D)**. *P ≤ 0.05, **P ≤ 0.01, ***, ^###^*P* ≤ 0.001.

To identify new inflammatory and apoptosis regulators in RA synoviocytes, genes induced by the pro-inflammatory cytokines TNF-α and IL-17A were systematically searched. To do so, a microarray dataset previously validated and derived from RA synoviocytes exposed for 12 h to TNF-α, IL-17A, or their combination was used. In this microarray, 130 genes were synergistically regulated by the combination of IL-17A and TNF (20, 21). Among these synergistically regulated genes, four genes were involved in apoptosis regulation. However, three of these genes were barely expressed in RA synoviocytes. The gene Amigo2 was therefore identified as the only apoptotic regulator sufficiently expressed and synergistically upregulated with the IL-17A/TNF combination (Figure 1A). This cytokine-mediated upregulation was specific to Amigo2 as compared to the other family member Amigo3, which did not get modulated by neither the cytokines alone nor their combination (Figure 1B). Amigo1 probeset was not present in the microarray dataset; therefore, its expression in inflammatory conditions could not be verified in RA synoviocytes.

The regulatory effect of IL-17A and TNF-α on Amigo2 expression was validated by RT-PCR and showed that the gene is already upregulated by TNF-α and IL-17A alone and that their combination synergistically upregulates its expression to up to sevenfold in RA synoviocytes (Figure 1C). In order to know whether this Amigo2 upregulation was specific to RA synoviocytes, its expression was also examined in synoviocytes originating from healthy donors and OA patients. In control situation, Amigo2 expression was higher in RA synoviocytes than in OA and healthy synoviocytes (Figure 1D). Furthermore, when cells were exposed to the IL-17A/TNF combination, a significant higher gene induction was observed in RA synoviocytes (ninefold) in comparison to both healthy and OA synoviocytes (fourfold and twofold, respectively, Figure 1D). These results demonstrate that Amigo2 induction in inflammatory conditions is more specific to RA synoviocytes.

### Coculture of RA Synoviocytes with Immune Cells Increases Amigo2 Expression in Both Cell Types

**Figure 2:**
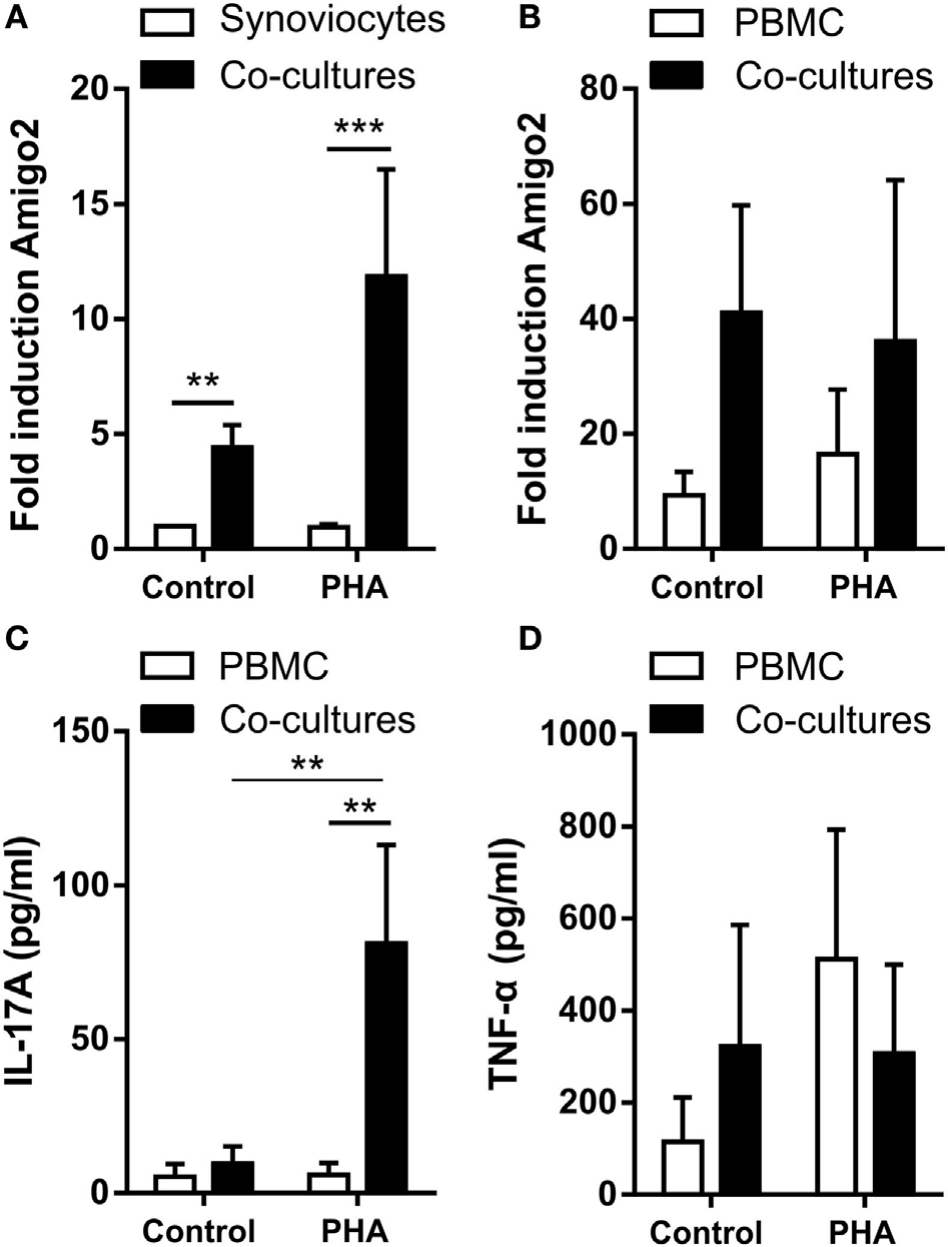
**Coculture of RA synoviocytes with immune cells increases Amigo2 expression in both cell types**. RA synoviocytes were cocultured with PBMC from healthy donors in the presence or not of PHA for 24 h. In the cocultures, PBMC were separated from synoviocytes by EDTA addition prior cell lysis. Amigo2 expression was assessed by quantitative real-time PCR and was expressed as fold changes compared to synoviocytes cultured alone and exposed to vehicle **(A,B)**. Amigo2 expression was evaluated in both synoviocytes **(A)** and PBMC **(B)** cultured alone or together. The production of IL-17A **(C)** and TNF-α **(D)** by the cocultures was quantified by ELISA. The production of these cytokines was not detectable in synoviocytes cultured alone. Data are the mean of at least three independent experiments ± SEM. *P ≤ 0.05, **P ≤ 0.01, ***P ≤ 0.001.

In order to better mimick the *in vivo* inflammatory situation where synoviocytes interact with immune cells, Amigo2 gene expression was also evaluated in RA synoviocytes cocultured with PBMC activated or not with PHA. Before lysis of the cells for RNA extraction, PBMC were separated from synoviocytes with EDTA in order to quantify Amigo2 expression only in the synoviocytes or the PBMC alone and not in the mixture of synoviocytes with PBMC. However, PBMC were only partially removed as measured by the expression of CD3, a marker present on the surface of T lymphocytes (Figure S1 in Supplementary Material). Coculture of RA synoviocytes with resting PBMC for 24 h already significantly induced Amigo2 gene expression to up to fivefold (Figure 2A). This Amigo2 induction was most likely triggered only by cell–cell contact since no significant IL-17A (Figure 2C) and TNF-α (Figure 2D) secretion occurred. In synoviocytes cocultured for 24 h with activated PBMC, Amigo2 gene expression was enhanced (up to 10-fold) (Figure 2A) with an increased secretion of IL-17A (Figure 2C). The production of TNF-α, however, did not change after PHA stimulation in comparison to the control situation (Figure 2D). Changes in amigo2 expression were seen in PBMC cocultured with RA synoviocytes in control condition with a high level of variability between PBMC donors (Figure 2B). PHA stimulation did not affect Amigo2 expression (Figure 2B) in PBMC cocultured with RA synoviocytes in comparison to the control situation. These results indicate that the cellular interactions between RA synoviocytes and immune cells trigger the induction of Amigo2 expression in both synoviocytes and PBMC.

### Amigo2 Induction in RA Synoviocytes Cocultured with Immune Cells Remains Stable Even after Immune Cell Removal

**Figure 3:**
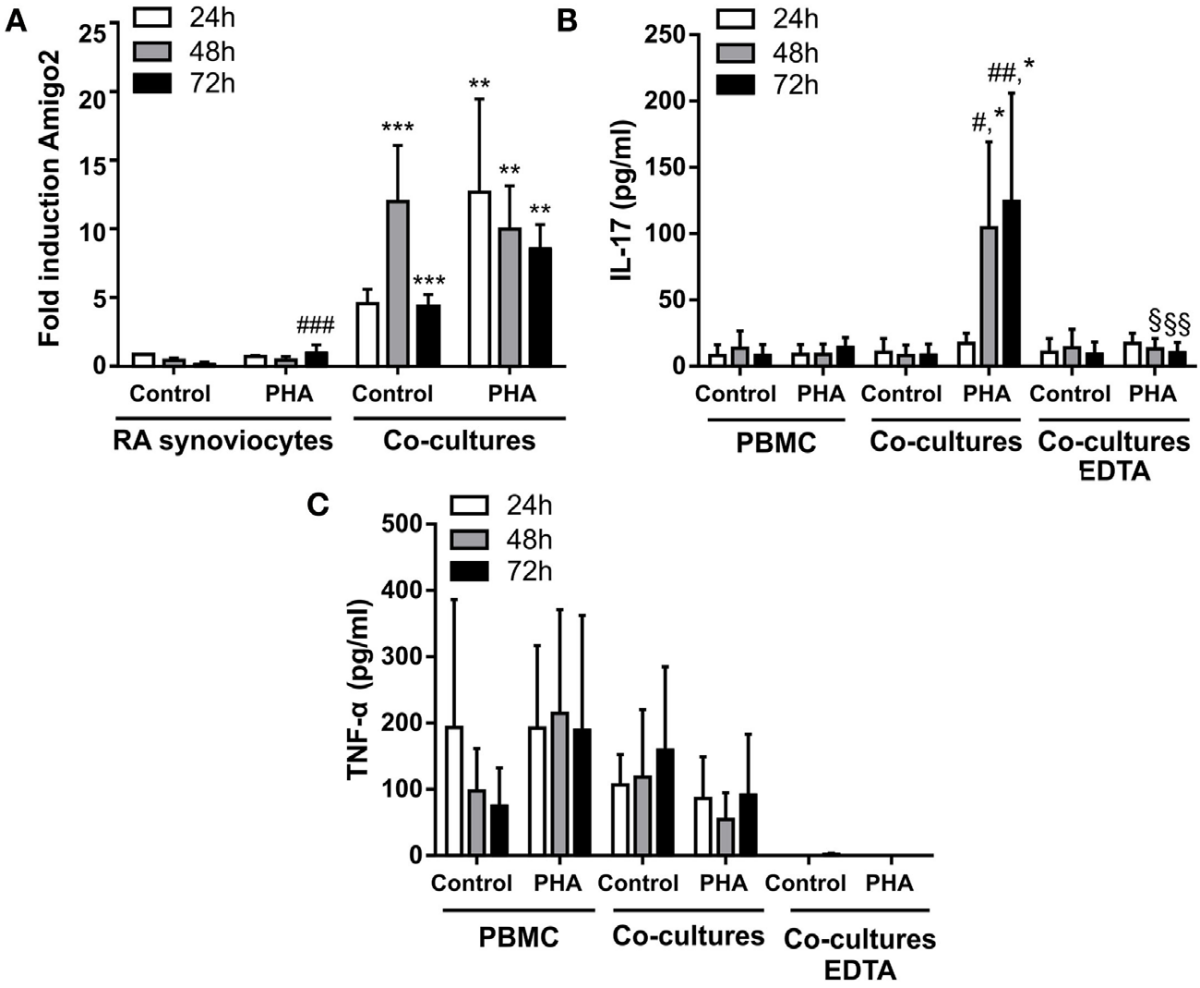
**Amigo2 induction in RA synoviocytes cocultured with immune cells remains stable even after immune cell removal**. RA synoviocytes were cocultured with PBMC from healthy donors in the presence or not of PHA for 24 h. In the cocultures, PBMC were separated from synoviocytes by EDTA addition. Synoviocytes were cultured for 24 or 48 h more after PBMC removal. Amigo2 expression was assessed at different time-points by quantitative real-time PCR and was expressed as fold changes compared to RA synoviocytes cultured alone and exposed to vehicle **(A)**. The production of IL-17A **(B)** and TNF-α **(C)** by the cocultures was quantified by ELISA. The production of these cytokines was measured in cocultures with no immune cell removal (cocultures) or with immune cell removal by EDTA (cocultures EDTA). Their production was not detectable in synoviocytes cultured alone. Data are the mean of at least three independent experiments ± SEM. ^#^comparison with control situation, *comparison between synoviocytes **(A)** or PBMC **(B)** alone and cocultures, ^§^comparison between cocultures with or without EDTA addition. *,#,§*P* ≤ 0.05, **,##,§§*P* ≤ 0.01, ***, ###*P* ≤ 0.001.

Since RA synoviocytes have been described to retain their aggressive phenotype months after removal from the RA synovial milieu (3, 22, 23), the induction of Amigo2 expression was evaluated in the cocultures over time after removing the immune cells at 24 h. When RA synoviocytes were cocultured with resting PBMC, Amigo2 expression was enhanced at 24 h to up to 5-fold and its expression continued to raise to more than 10-fold even after partial PBMC removal (Figure 3A). This increase in Amigo2 expression occurred despite the barely detectable levels of both IL-17A (Figure 3B) and TNF-α (Figure 3C) after partial PBMC removal. However, at 72 h Amigo2 expression dropped back to the levels observed at 24 h (fivefold, Figure 3A). In the cocultures of RA synoviocytes with activated PBMC, Amigo2 expression increased to almost 15-fold after 24 h and slowly decreased over time after PBMC removal (Figure 3A). At 72 h, Amigo2 expression levels had a tendency although not significant to remain higher than the ones observed with the cocultures performed with resting PBMC (10-fold versus 5-fold, respectively, Figure 3A). These results indicate that induction of Amigo2 expression in RA synoviocytes that have been in contact with activated immune cells remains even in the absence of the cellular interaction and the inflammatory environment.

### Amigo2 Expression Is Regulated by MAPKs and HMGB1 in Inflammatory Conditions

**Figure 4:**
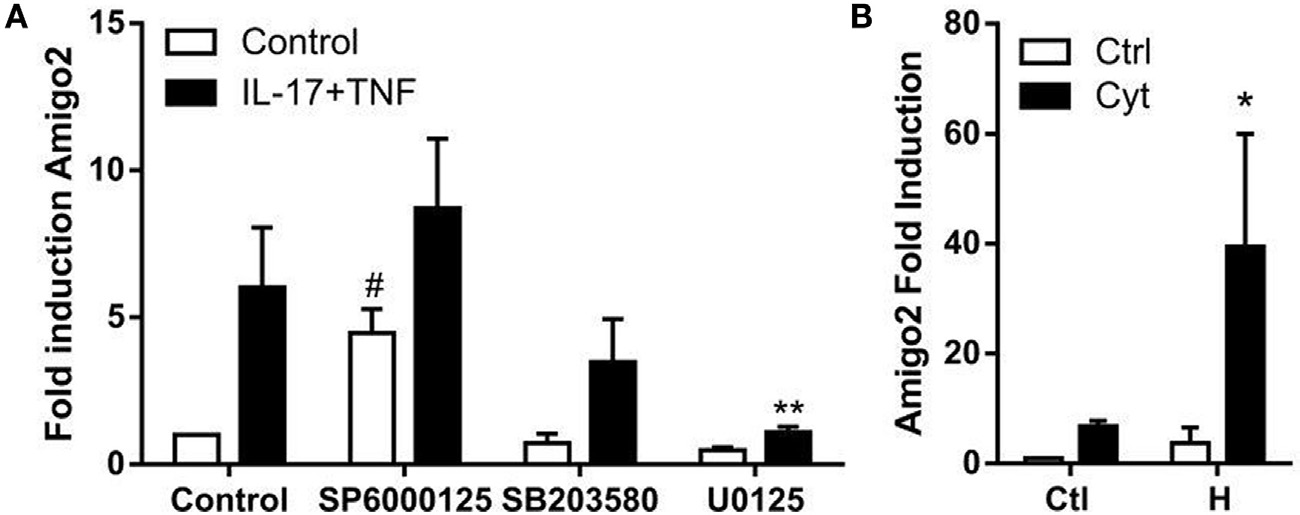
**Amigo2 expression is regulated by MAPKs and HMGB1**. RA synoviocytes were preexposed to MAPKs inhibitors for 1 h followed by the addition of a combination of IL-17A and TNF-α **(A)** or co-exposed to HMGB1 and the TNF/IL-17A combination **(B)**. After 12 h, Amigo2 expression was assessed by quantitative real-time PCR and is expressed as fold changes compared to control situation. SP6000125, JNK inhibitor; SB203580, p38 inhibitor; U0125, ERK inhibitor; Ctl, control; Cyt, cytokines; H, HMGB1. Data are the mean of at least three independent experiments ± SEM. ^#^Comparison with control situation, *comparison with cytokine-treated cells. *,^#^*P* ≤ 0.05, **P ≤ 0.01.

Since nothing is known yet about the regulation of Amigo2 expression in synoviocytes, the implication of several potential regulators was investigated. It was previously reported that MAPKs are involved in the regulation of several processes in RA synoviocytes including apoptosis (24), and their role in the signaling cascade of Amigo2 is yet unknown. Therefore, the effect of the three MAPKs c-jun N-terminal kinase (JNK), extracellular signal regulated kinase (ERK), and p38 on Amigo2 expression was examined by inhibiting them 1 h prior the addition of cytokines. JNK inhibition led to a significant induction in Amigo2 expression already in control conditions (fourfold), which was enhanced to up to eightfold in the presence of cytokines (Figure 4A). On the contrary, ERK inhibition led to a decrease in Amigo2 expression in control condition and completely abrogated the cytokine-mediated induction in Amigo2 expression (Figure 4A). p38 inhibition did not affect Amigo2 expression (Figure 4A), indicating that it is not involved in the regulation of its expression. These results demonstrate that JNK and ERK regulate Amigo2 expression in opposite manners with JNK acting as an inhibitor of Amigo2 expression and ERK acting as an activator.

The related family member AMIGO was first discovered in a systematic screen searching for genes induced on HMGB1-coated matrix (15). Since HMGB1 has been implicated in RA pathogenesis, the regulation of Amigo2 by HMGB1 was investigated in RA synoviocytes. Amigo2 expression was therefore quantified after exposure of the cells for 12 h to HMGB1 alone or in combination with IL-17A and TNF-α. HMGB1 alone increased Amigo2 expression to more than threefold (Figure 4B). Furthermore, the combination of both HMGB1 and cytokines led to a significant synergistic induction (39-fold, Figure 4B). These results demonstrate that alike the closely related family member Amigo, Amigo2 expression is regulated by HMGB1, and that HMGB1 can synergize with cytokines to further enhance its expression.

### Amigo2 Expression Levels Correlate with Cell Death

**Figure 5:**
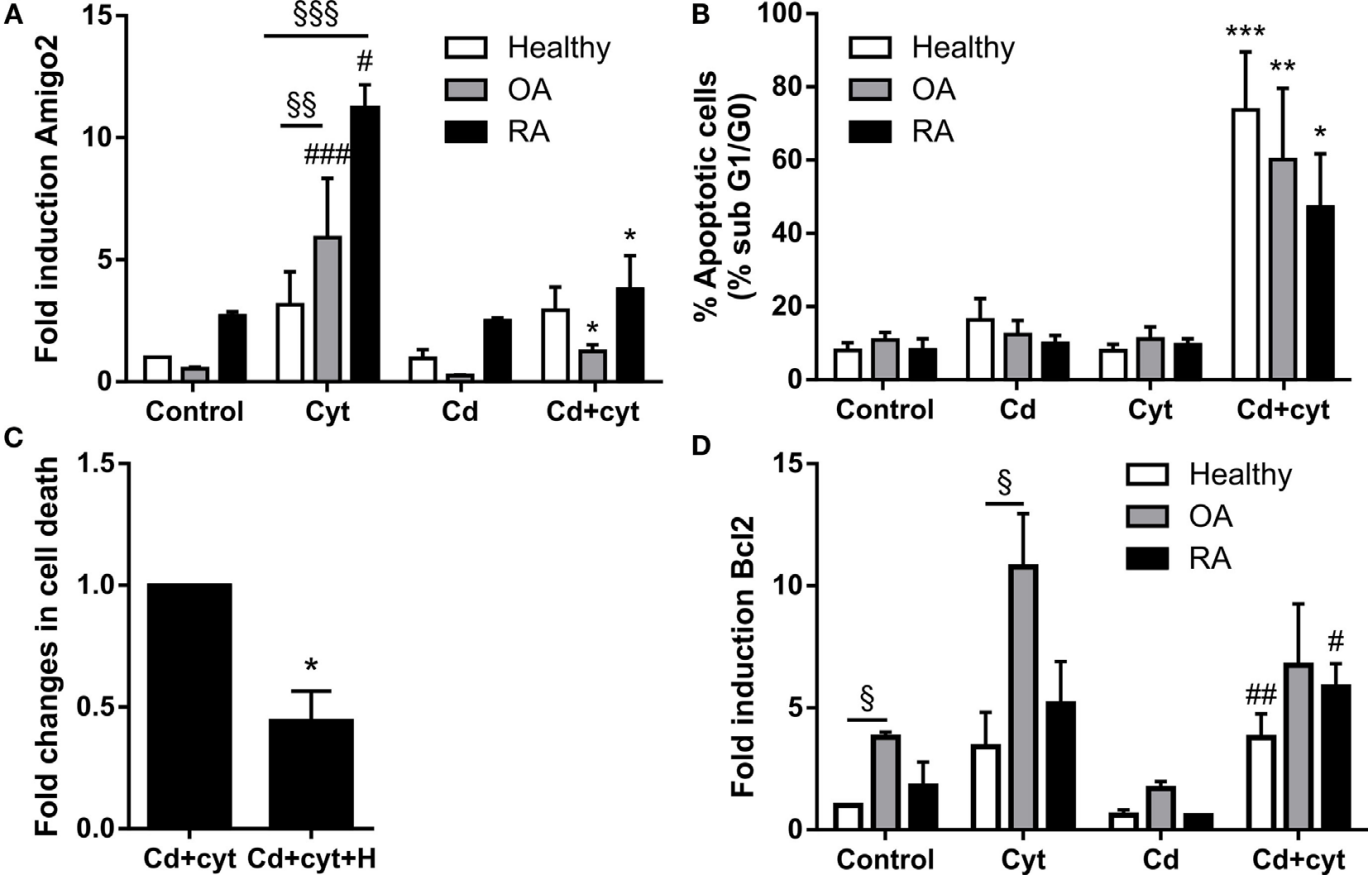
**Amigo2 expression levels correlate with cell death**. Healthy, OA and RA synoviocytes were preexposed to a combination of IL-17A and TNF-α overnight before addition of a low dose of the proapoptotic agent Cd **(A,B,D)**. Amigo2 expression was then evaluated 6 h after Cd addition by quantitative real-time PCR and is expressed as fold changes compared to healthy synoviocytes exposed to vehicle **(A)**. Cell death was determined by cell cycle analysis after a week **(B)**. To evaluate the effect of HMGB1 on cell death, RA synoviocytes were preexposed to a combination of IL-17A and TNF-α in the presence or not of HMGB1 followed by Cd addition. Cell death was quantified by cell cycle analysis after a week and is represented as fold changes compared to cells treated with Cd and cytokines **(C)**. Similarly to Amigo2, Bcl2 expression was evaluated by quantitative real-time PCR and is expressed as fold changes compared to healthy synoviocytes exposed to vehicle **(D)**. Data are the mean of at least three independent experiments ± SEM. Cyt, cytokines; H, HMGB1. ^#^Comparison with control situation, *comparison with cytokine treated cells, ^§^comparison between cell types. *,#*P* ≤ 0.05, **,§§*P* ≤ 0.01, ***,###,§§§*P* ≤ 0.001.

Since Amigo2 is involved in the survival of other cell types (20, 21), the correlation between its expression and the apoptosis outcome of the cells was investigated. Synoviocytes from different clinical settings were treated with a combination of TNF-α and IL-17A followed by their exposure to a low dose of the cytotoxic agent cadmium (Cd). Preliminary experiments indicated that Cd could induce significant apoptosis at concentration as low as 0.1 ppm in inflammatory conditions. Amigo2 gene expression was then evaluated after a 6-h exposure to Cd (Figure 5A), a time point at which cell death did not yet occur, and cell death was evaluated after a week (Figure 5B). As demonstrated before, Amigo2 induction with cytokines was significantly higher in RA synoviocytes than in healthy and OA synoviocytes (Figure 5A). Exposure of the synoviocytes to Cd alone did not affect Amigo2 expression (Figure 5A) and did not induce any significant cell death (Figure 5B). Interestingly, Cd significantly inhibited the cytokine-mediated Amigo2 induction in both OA and RA synoviocytes (Figure 5A). This corroborated with an increased cell death in cells exposed to Cd in inflammatory conditions (Figure 5B). Healthy synoviocytes presented a slight Amigo2 induction with cytokines, which was not affected by Cd (Figure 5A). However, Amigo2 expression levels were as low as the ones observed in OA and RA synoviocytes, which could explain their sensitivity to Cd-induced toxicity in inflammatory conditions (Figure 5B). These results indicate that cell death correlates with the expression levels of the antiapoptotic gene Amigo2 in synoviocytes. The involvement of Bcl2 in the cell death outcome was also explored. Bcl2 expression was increased significantly with the cytokine combination only in OA synoviocytes (Figure 5D). However, no difference was observed in Bcl2 expression in synoviocytes from the different clinical settings when exposed to Cd in the presence of cytokines (Figure 5D). These results indicate that Bcl2 is not involved in the resistance to Cd-induced apoptosis in inflammatory conditions.

Since Amigo2 expression levels correlated with cell death, the effect of HMGB1 in inflammatory conditions was also evaluated at the cell death level. RA synoviocytes were preexposed to HMGB1 alone or in combination with cytokines overnight followed by Cd addition, and cell death was evaluated after a week. Addition of HMGB1 is reduced by half the Cd-induced apoptosis in inflammatory conditions (Figure 5C). These data indicate that HMGB1 protects RA synoviocytes against Cd-induced apoptosis in inflammatory conditions in part by upregulating Amigo2 expression.

## Discussion

Despite the variety of current treatments in use for RA, none of them completely cure it but rather slow the progression of the symptoms. Indeed, RA pathogenesis is complex and involves many cell types including several immune cells as well as the resident fibroblasts. These cells play a key role by proliferating excessively and by producing cytokines that perpetuate inflammation and proteases that contribute to cartilage destruction. In the present study, we systematically searched for genes controlling inflammation and apoptosis regulated by the two pro-inflammatory cytokines IL-17A and TNF-α in synoviocytes. Using this approach, we identified the novel antiapoptotic regulator Amigo2.

We demonstrated that Amigo2 expression is regulated by several players in RA synoviocytes. First, the combination of IL-17A and TNF-α synergistically increased its expression specifically in RA synoviocytes. These two cytokines are already known to synergize to induce the production of several pro-inflammatory mediators including IL-6, IL-1β, and IL-8 as well as antiapoptotic molecules such as synoviolin in RA synoviocytes (4). The underlying molecular mechanisms of such synergy are still unclear. One explanation is the ability of IL-17A to stabilize posttranscriptionally the mRNA of some of the TNF-induced genes (25–29). Another explanation is the enhancing effect of the IL-17A/TNF combination on the expression of the TNF type II receptor (20). It is not sure which of these mechanisms controls the synergistic effect observed on Amigo2 expression.

We also showed that Amigo2 expression was regulated in opposite manner by the two MAPKs, JNK and ERK. ERK promoted Amigo2 expression while JNK suppressed it. It has been previously showed that ERK predominantly regulates RA synoviocytes proliferation (30) while JNK controls the expression of matrix metalloproteinase 3 (MMP3) in RA synoviocytes and thereby joint destruction (31). Therefore, it seems that Amigo2 expression is tightly regulated by these two MAPKs and we could speculate that a small shift in this tight regulation could drive the cells toward a specific fate. Increased Amigo2 expression would lead to excessive proliferation whereas decreased Amigo2 expression would promote the invasive phenotype of the cells.

In addition to the MAPKs, we demonstrated that cell–cell contact between immune cells and the synoviocytes promoted Amigo2 expression in both cell types. This induction was further enhanced when the immune cells were activated by PHA. We previously revealed that interaction of PBMC with RA synoviocytes promoted the activation and expansion of Th17 cells through caspase 1 activation (32). Here, we show for the first time that cell–cell contact promotes also the upregulation of antiapoptotic molecules such as Amigo2. Since the cocultures were performed with PBMC, containing various types of immune cells, it cannot be sure which immune cells express the most Amigo2. It could be that several of them express the gene following the example of the antiapoptotic mediator synoviolin expressed in both Th17 cells and B cells (9). However, this remains to be determined. Interestingly, the induction in Amigo2 expression persisted in RA synoviocytes even after the partial removal of immune cells. It was already shown that RA synoviocytes present imprinted anomalies, such as mutations or epigenetic changes (8), and that they can destroy human cartilage many months after removal from the RA synovial milieu when engrafted into mice with severe combined immunodeficiency (SCID) (19, 25). Here, we show that the upregulation in Amigo2 expression after cell–cell contact with activated immune cells can be maintained at least until more than 72 h. We can therefore speculate that the constant cellular interactions between the synoviocytes and the immune cells infiltrating the synovium maintain high levels of Amigo2 in the synoviocytes of RA patients.

Finally, we showed that Amigo2 expression is synergistically upregulated by the IL-17A/TNF combination and the heparin-binding protein HMGB1. HMGB1 is known to be implicated in RA pathogenesis. It is present in excessive levels in joints and serum of RA patients, and antagonistic HMGB1 therapies ameliorate arthritis in murine models (18). HMGB1 induces synergistic interactions by forming complexes with certain other pro-inflammatory molecules such as lipopolysaccharide (LPS) or IL-1β (33). In this study, we showed for the first time that HMGB1 also enhances the effect of IL-17A and TNF-α in synoviocytes. This enhancing effect is most likely indirect *via* the activation of the toll-like receptors (TLRs) which, in turn, activate the transcription factor nuclear factor κ B (NF-κB) leading to the transcription of more TNF-α (18).

**Figure 6:**
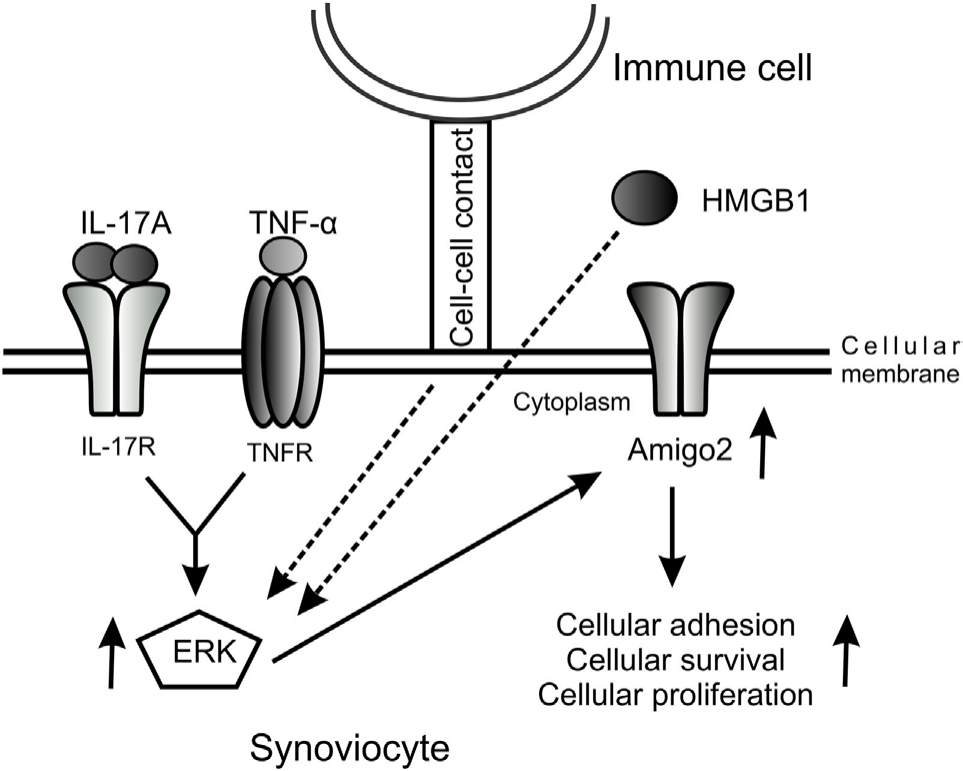
**Model of the regulation of Amigo2 expression which promotes cell survival and proliferation**. A model was proposed whereby cellular interaction with immune cells and exposure of the cells to the pro-inflammatory cytokines IL-17A and TNF-α as well as to HMGB1 increases Amigo2 expression in an ERK-dependent manner leading to enhanced cellular adhesion and promoting cell survival and proliferation.

Furthermore, we demonstrated in this study that Amigo2 expression levels correlated with the cellular outcome of the cells. Indeed, when cells were exposed to the cytotoxic agent Cd in inflammatory conditions, Cd inhibited the IL-17A/TNF-mediated induction in Amigo2, which corroborated with an increased apoptosis. Moreover, the increase in Amigo2 expression by the HMGB1/IL-17-A/TNF combination was correlated with a cellular protection against cd-induced toxicity. However, the direct effect of Amigo2 on cell survival remains to be proven. siRNA-mediated knockdown of Amigo2 in RA synoviocytes only led to a 25% knockdown efficiency in our hands and did not affect the cellular outcome of the cells (data not shown). AMIGO2 is a transmembrane protein known to form homophilic and heterophilic interactions with other AMIGO family members (21). It is possible that the other members of the family could compensate when Amigo2 is partially depleted. Amigo3 was not induced by IL-17A and TNF in RA synoviocytes. However, the expression of Amigo1 could not be verified as its corresponding probeset was not present in the microarray dataset used. Therefore, Amigo1 could potentially compensate for Amigo2 once depleted. Since Amigo2 is an adhesion molecule, we believe that an increase in its expression leads to an enhancement in the cell–cell contacts protecting the cells against cellular stress and hypoxia and promoting cellular proliferation. We suggest a model whereby cell–cell contact with immune cells and the presence of pro-inflammatory cytokines and HMGB1 in the joints of RA patients promote Amigo2 expression in an ERK-dependent manner and enhance their resistance to apoptosis (Figure 6).

## Author Contributions

GB: conception of the work, acquisition and interpretation of the data, and elaboration of the manuscript; PB and FL: acquisition of data, revising, and approval of the article; PM: conception of the work, interpretation of the data, critical revision, and final approval of the article.

## Conflict of Interest Statement

The authors declare that the research was conducted in the absence of any commercial or financial relationships that could be construed as a potential conflict of interest.
