# Supplementary figures and images for: IL-17A and TNF-α Increase the Expression of the Antiapoptotic Adhesion Molecule Amigo-2 in Arthritis Synoviocytes
Source: Front Immunol. 2016 Jun 27;7:254. doi: 10.3389/fimmu.2016.00254 (PMC4922130; doi:10.3389/fimmu.2016.00254)

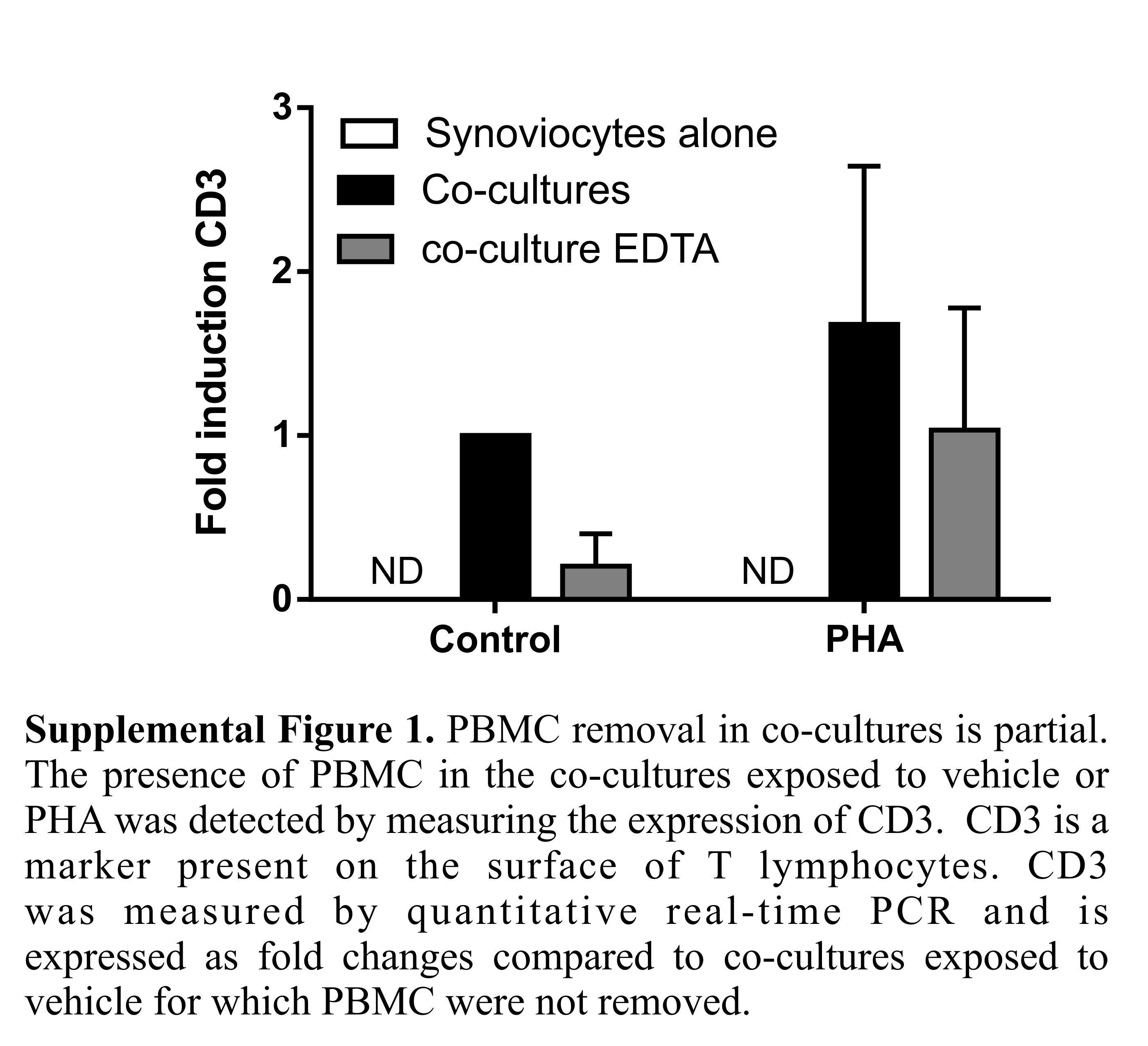

Supplement: Supplementary file 1 [file Image_1.tif]
